# Leveraging language models to categorize individual‐level priorities and deliver personalized brain health recommendations

**DOI:** 10.1002/alz70858_103890

**Published:** 2025-12-26

**Authors:** Claudio Toro‐Serey, Stina Saunders, Marissa C Ciesla, Seng Oon Toh, Alvaro Pascual‐Leone, Sean Tobyne

**Affiliations:** ^1^ Linus Health, Boston, MA, USA; ^2^ Linus Health Europe, Dublin, Ireland; ^3^ Hebrew SeniorLife, Boston, MA, USA; ^4^ Harvard Medical School, Boston, MA, USA

## Abstract

**Background:**

Cognitive impairment affects many aspects of a person's life. While new treatments can slow down cognitive decline, this alone might not directly preserve a person's ability to engage in what matters most to them. Identifying an individual's brain health priorities and tailoring actionable recommendations to preserve them can produce meaningful treatment benefits and outcome measures to track treatment efficacy. We have previously described a scalable, self‐administered digital tool for tracking individually defined priority outcomes, the electronic person specific outcome measure (ePSOM). Here we used language models to analyze free text responses and discover common themes to 1) examine the degree of heterogeneity in individual brain health priorities and 2) evaluate algorithms that automatically link unseen free text responses to these themes.

**Methods:**

We clustered 16,000 ePSOM free‐text responses from 780 individuals in the US. First, we used data augmentation procedures to transform each sentence into a slightly different phrase with similar semantic meaning. Each sentence from the original and augmented sets was embedded into a vector with 384 dimensions using a sentence transformer architecture. We evaluated clustering solutions by calculating 1) the similarity in clusters between sets (intersample similarity, using the adjusted rand index, where 0 is chance and 1 is complete agreement); and 2) the proportion of augmented sentences that had the highest average cosine similarity to the cluster containing their original counterpart (assignment similarity).

**Results:**

A hierarchical clustering approach yielded the best results, showing that 545 clusters had the highest intersample (0.56) and assignment (0.75) similarities. Assignment similarity increased to 0.90 when considering the top three most similar original clusters to each augmented sentence.

**Conclusions:**

There is heterogeneity in what matters to individuals in the context of cognitive health. Person‐specific priorities should be taken into account when planning treatments. We show that using language models, we are able to identify clusters which most closely align with individual free form responses. By creating educational materials and behavioral interventions tied to these clusters, we can eventually provide personalized recommendations at scale to sustain brain health and reduce the risk of brain‐related disabilities (e.g. dementia).

